# Dwarfs under dinosaur legs: a new millipede of the order Callipodida (Diplopoda) from Cretaceous amber of Burma

**DOI:** 10.3897/zookeys.841.34991

**Published:** 2019-05-02

**Authors:** Pavel Stoev, Leif Moritz, Thomas Wesener

**Affiliations:** 1 National Museum of Natural History, Sofia, Tsar Osvoboditel Blvd. 1, Sofia 1000, Bulgaria National Museum of Natural History Sofia Bulgaria; 2 Pensoft Publishers, Sofia, Bulgaria Pensoft Publishers Sofia Bulgaria; 3 Zoological Research Museum Alexander Koenig (ZFMK), Leibniz Institute for Animal Biodiversity, Adenauerallee 160, D-53113, Bonn, Germany Zoological Research Museum Alexander Koenig, Leibniz Institute for Animal Biodiversity Bonn Germany

**Keywords:** Burmanopetalidea suborder nov., Burmanopetalidae fam. nov., *Burmanopetaluminexpectatum* gen. nov. et sp. nov., Cenomanian, Mesozoic.

## Abstract

The entire Mesozoic Era is rather poor in millipede (class Diplopoda) fossils, with less than a dozen species being taxonomically described. Here, we describe the first fossil millipede of the order Callipodida, *Burmanopetaluminexpectatum***gen. nov. et sp. nov.**, found in early Cenomanian amber of Burma, 98.79±0.62 Mya. The species possesses a number of morphological traits that exclude it from all extant suborders, and Burmanopetalidea suborder nov. and Burmanopetalidae fam. nov. are here erected to accommodate it. The new suborder can be recognized by the following unique characters: pleurotergal setae absent; telson with a specific spatulate shape twice the size of the penultimate body ring; hypoproct devoid of setae; and eyes composed of five well-separated ommatidia. While the callipodidan habitus seems to have remained generally unchanged for at least 99 million years, pleurotergal and hypoproctal setation, as well as the complexity of eyes in ground-dwelling forms may have evolved recently in the order. As *B.inexpectatum***gen. nov. et sp. nov.** is the first true callipodidan in the fossil record, the minimum age of Callipodida is thus at least 99 Mya.

## Introduction

Millipedes (Diplopoda) are a highly diverse but also a largely understudied group of arthropods with >11,000 described species (Enghoff et al. 2015). The actual number of species is estimated to be between 15,000–20,000 (Brewer et al. 2012) or 50,000–80,000 (Minelli and Golovatch 2013). The earliest fossil records of millipedes come from the Middle Silurian or Lower Devonian of Scotland about 420 Mya, where three archipolypod species were found (Wilson and Anderson 2004; Shear and Edgecombe 2010; Wolfe et al. 2016; Suarez et al. 2017). Being the first animals to conquer land (Wilson and Anderson 2004), millipedes play a significant ecological role as major destruents in the terrestrial ecosystems probably since the Silurian (Kime and Golovatch 2000; Golovatch and Kime 2009).

Callipodida is a small order of spinneret-carrying millipedes of the superorder Nematophora (Blanke and Wesener 2014; Enghoff et al. 2015). The exact relationship with the other two nematophoran orders, Stemmiulida and Chordeumatida, is not yet clarified. Callipodida are considered a sister-group either to Stemmiulida (Blanke and Wesener 2014) or to Chordeumatida (Brewer and Bond 2013). Some callipodidans are among the handful of known carnivorous species of the Diplopoda (Hoffman and Payne 1969). In addition, several species of Callipodida are unusually fetid due to their defense secretions containing *p*-cresol (Makarov et al. 2011), and can be smelled several meters away (author observations).

Callipodida show a disjunct distribution in the Northern Hemisphere, with three major centers of diversification – the North Mediterranean region, Central and South East Asia, and North America. The order is absent from South America, Africa, the Pacific Islands, Australia, and the northern parts of Eurasia (Shear et al. 2003; Shelley and Golovatch 2011). The group is also remarkably absent from the Indian subcontinent (Golovatch and Wesener 2016), with just one species, *Bollmaniakohalana* (Attems, 1936), from the region of Kashmir, between Pakistan and India (Stoev et al. 2008). Despite their interesting distribution pattern, the phylogenetic relationships within Callipodida are largely unresolved, which also holds true for all but five millipede orders (Simonsen 1990; Enghoff 1991; Wesener and VandenSpiegel 2009; Pitz and Sierwald 2010; Oeyen and Wesener 2018). The order is moderately rich, with around 140 known extant species (Stoev et al. 2008) grouped into three suborders, eight families, and 36 genera or subgenera (Enghoff et al. 2015).

While the known Paleozoic millipedes significantly differ from extant forms and the Cenozoic fossils can be placed in extant families and genera, the fossil record of millipedes in the entire Mesozoic Era was considered extremely poor (see Shear 1998; Shear and Edgecombe 2010; Edgecombe 2015) until recent discoveries in Burmese amber (Liu et al. 2017a; Wesener and Moritz 2018; Jiang et al. 2019).

Cockerell (1917) was the first to describe a millipede from Burmese amber, *Polyxenusburmiticus* Cockerell, 1917, a species which was later transferred to the extant genus *Phryssonotus* Scudder, 1885 (Rasnitsyn and Golovatch 2004; Zhang 2017). Just recently, two species of Siphoniulida (Liu et al. 2017a) and one species of Siphonophorida (Jiang et al. 2019), all belonging to extant genera, have been added to the list. So far there is no described non-amber fossil which can be definitely placed in the order Callipodida, but Shear et al. (2009) hypothesized that *Hannibaliuluswilsonae*Shear, Selden & Gall, 2009 from the Triassic of France could be a representative of Callipodida, although clear apomorphies of the order were not observable.

Burmese amber from the Hukawng Valley in Kachin State, northern Myanmar (formerly Burma), is precisely dated to the Cretaceous Cenomanian 98.79±0.62 Mya (Shi et al. 2012) and has a long history of exploitation. For a review of its history and geology see Zherikhin and Ross (2000), Grimaldi et al. (2002), and Cruickshank and Ko (2003). Burmese amber has proven to be an important source of arthropod fossils, containing no less than 849 described species of arthropods (Ross et al. 2010; Ross 2018). Recently, the great importance of Burmese amber for the understanding of the Myriapoda fossil record and historical biogeography was demonstrated with the discovery of two species of the enigmatic order Siphoniulida (Liu et al. 2017a), a species of the order Siphonophorida (Jiang et al. 2019), and the first known fossil representative of the Symphyla family Scolopendrellidae (Moritz and Wesener 2018). A recent investigation of 460 newly discovered Diplopoda inclusions in Burmese amber included specimens belonging to 13 of the 16 extant orders, as well as the oldest known fossil representatives for eight extant orders (Wesener and Moritz 2018). Among the 529 millipede specimens hitherto known from Burmese amber (Wesener and Moritz 2018; Jiang et al. 2019), a single female specimen belongs to the order Callipodida, and its description is the purpose of this paper.

## Material and methods

### Material and data deposition

The single female specimen (ZFMK-MYR07366) came into our possession from the private collection of Mr Patrick Müller and is deposited in the Zoological Research Museum A. Koenig (ZFMK, Bonn, Germany). The authenticity of the amber was checked under UV light, producing a characteristic pale blue colour when photographed (Xing et al. 2016). All legal exportation permits were obtained and are available upon request. µCT-data are deposited in MorphoBank (O'Leary and Kaufman 2012) under project number 3360 (http://morphobank.org/permalink/?P3360).

### Light microscopy and photography

Morphological characters were investigated with a Discovery.V12 stereo-microscope (Zeiss) and a BX51 light microscope (Olympus). Photographs were taken with a Canon EOS 7D camera equipped with magnifier lenses.

### Micro-computer tomography (µCT) and visualization

µCT-Scans were acquired with a SKYSCAN 1272 (Bruker microCT, Kontich, Belgium) at the Zoological Research Museum Alexander Koenig. For the parameters, see the media information in MorphoBank (http://morphobank.org/permalink/?P3360). Thermal-drift correction, ring artefact reduction and digital section reconstruction was done with NRecon 1.7 (Bruker microCT, Kontich, Belgium). Volume rendering and measurements were performed in Drishti version 2.6.3 (Limaye 2012).

### Terminology

We use ‘body ring’ when pleurotergites and sterna are referred to collectively. Callipodida do not have fused rings as in some other millipede orders (eg. Julida) because the sterna are free.

## Results

### Systematic palaeontology

#### Class Diplopoda de Blainville in Gervais, 1844

##### Subclass Chilognatha Latreille, 1802/1803

###### Infraclass Helminthomorpha Pocock, 1887

####### Superorder Nematophora Verhoeff, 1913

######## Order Callipodida Pocock, 1894

######### Suborder † Burmanopetalidea

Taxon classificationAnimaliaCallipodida

suborder nov.

########## Diagnosis.

Body less than 10 mm, composed of 35 body rings (including collum and two apodous body rings) and telson. Eyes composed of five ommatidia situated in two rows (3+2). Body rings cylindrical, with fused tergites and pleurites and free sternites. Pleurotergites composed of smooth prozonites and carinate metazonites, latter being greater in diameter than prozonites. Pleurotergal crests most pronounced from 3^rd^ to 8^th^ pleurotergite. Pleurotergal setae absent; telson spatulate, twice the size of the penultimate body ring; hypoproct tripartite, devoid of setae.

The suborder comprises one family: †Burmanopetalidae fam. nov.

######### Family † Burmanopetalidae fam. nov.

Taxon classificationAnimaliaCallipodidaBurmanopetalidae

http://zoobank.org/37E00121-783A-4842-961D-5DB98ADA1BDD

########## Diagnosis.

As for the suborder.

########## Type genus.

†*Burmanopetalum* gen. nov.

######### Genus † Burmanopetalum gen. nov.

Taxon classificationAnimaliaCallipodidaBurmanopetalidae

http://zoobank.org/DB4A75E7-8626-4B8B-B172-43E2D7D51D71

########## Type species.

†*Burmanopetaluminexpectatum* sp. nov.

########## Etymology.

From “Burma”, the country of origin, and “-petalum” a frequent generic termination in Callipodida. Gender: neuter.

########## Diagnosis.

Differs from all extant genera of Callipodida by its minute size (less than 1 cm in length), lack of pleurotergal setae, and its spatulate telson being twice the size of the penultimate body ring. Eyes composed of five ommatidia.

######### † Burmanopetalum inexpectatum sp. nov.

Taxon classificationAnimaliaCallipodidaBurmanopetalidae

http://zoobank.org/DC6B3267-B386-4C1B-9D0A-7404AF469D32

**Figure 1.**
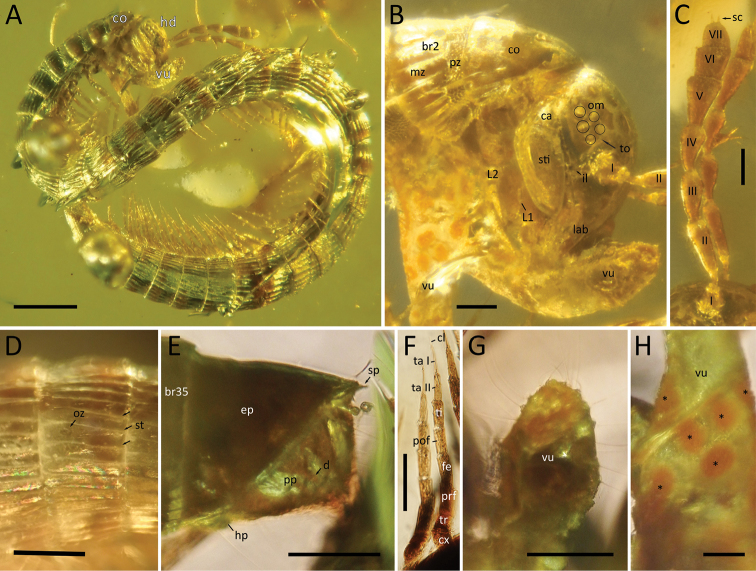
*Burmanopetaluminexpectatum* gen. nov. et sp. nov., female holotype (ZFMK-MYR07366) **A** habitus **B** head, anterior-most body rings and vulvae, anterior view **C** antennae, lateral view **D** pleurotergal crests ornamentation, lateral view **E** telson, lateral view **F** legs, dorsolateral view **G** apical part of vulva, lateral view **H** basal part of vulva, lateral view. Abbreviations: I–VII = antennomeres I–VII; br# = body ring number #; ca = mandibular cardo; cl = claw; co = collum; cx = coxa; d = division of paraproct; ep = epiproct; fe = femur; hd = head; hp = hypoproct; il = incisura lateralis; L# = leg number #; lab = labrum; mz = metazonite; om = ommatidia; oz = ozopore; pof = postfemur; pp = paraproct; prf = prefemur; pz = prozonite; sc = sensory cones; sp = spinnerets; st = stria; sti = mandibular stipes; ta I = tarsus I, ta II = tarsus II; ti = tibia; to = Tömösváry organ; tr = trochanter; vu = vulva; * = reddish circles of the basal part of vulvae. Scale bars: 500 µm (**A**), 100 µm (**B–G**).

**Figure 2.**
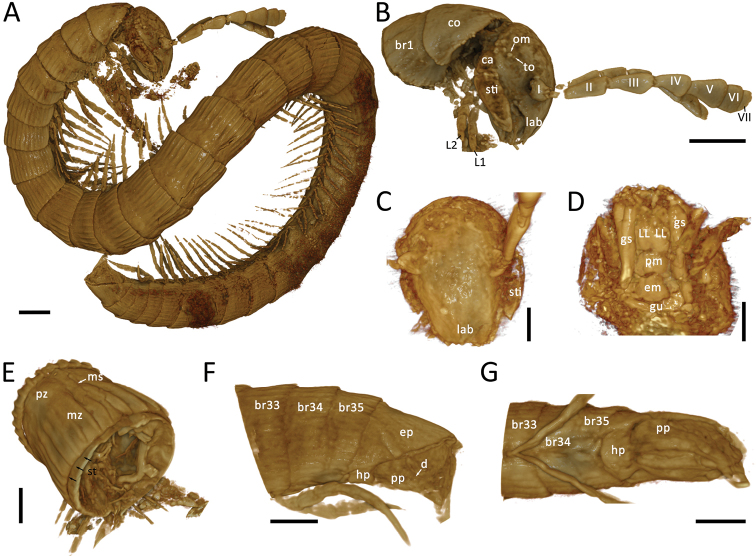
*Burmanopetaluminexpectatum* gen. nov. et sp. nov., female holotype (ZFMK-MYR07366), volume rendering **A** habitus **B** head, collum and pleurotergite 2, lateral view **C** head, anterior view **D** Gnathochilarium, ventral view **E** midbody body ring, dorsoposterior view **F** telson, and the last 3 pleurotergites, lateral view **G** same, ventral view. Abbreviation: I–VII = antennomere; br# = body ring number #; ca = mandibular cardo; co = collum; d = division of paraproct; em = eumentum; ep = epiproct; gs = gnathochilarium stipes; gu = gula; hp = hypoproct; lab = labrum; LL = lamella lingualis; L# = leg number #; ms = median suture; mz = metazonite; om = ommatidia; pm = promentum; pp = paraproct; pz = prozonite; sti = mandibular stipes; to = Tömösváry organ. Scale bars: 200 µm (**A**), 100 µm (**B–F**).

Figures 1A–H , 2A–G , 3

########## Previous records.

Callipodida, family undetermined: Wesener and Moritz 2018: 1135–1136, fig. 2C.

########## Material examined.

Holotype (ZFMK-MYR07366), from the collection of Mr Patrick Müller (transferred to ZFMK), adult female, Myanmar, Kachin State, Hukawng Valley, Noije Bum amber mine, 26°15'N, 96°34'E.

########## Diagnosis.

As for the suborder, family and genus. Species further characterized by antennomeres III–V strongly conical (infundibular), VI and VII subrectangular; metazonites with 28 more or less well-developed narrow, subparallel crests, well-separated from one another, poriferous crests missing.

########## Etymology.

"inexpectatum" in Latin means "unexpected" referring to the stunning discovery of just a single specimen among the 529 millipede specimens so far found in Burmese amber. The species epithet is an adjective.

########## Locality and horizon.

Burmese amber, early Cenomanian, 98.79±0.62 Mya (Shi et al. 2012) from the Noije Bum amber mine, Hukawng Valley, Kachin State, northern Myanmar.

########## Taphonomic features.

***Amber*** : Cut and polished. Piece rectangular, upper surface slightly convex, 14.1 mm × 6.3 mm × 2.5 mm. Colour: light yellow transparent.

***Specimen*** : Close to surface, body coiled in S-shape, vulvae extended.

***Syninclusions*** : Ensifera (Insecta: Orthoptera), Stellate hairs, large grayish spherical structure (Sporangia?).

########## Description.

Body length: 8.2 mm (measured from the CT scan); width of largest body ring 14: 0.4 mm. Body composed of 35 body rings and telson (Figs 1A, 2A, 3).

**Figure 3.** *Burmanopetaluminexpectatum* gen. nov. et sp. nov., female holotype (ZFMK-MYR07366), 3D model, volume rendering.

***Head*** elliptical, longer than wide, covered by long setae (Figs 1B, 2B,C). Labrum with 3 teeth. Eyes composed of 5 ommatidia situated in 2 rows (3+2). Incisura lateralis present, extending from mandible stipes to antennal base. Antennae (Fig. 1C) long and slender reaching to, or slightly extending beyond posterior margin of body ring 4 when folded backwards. Antennae 0.9 mm long, relative antennomere lengths II>V>III>IV>VI>VII=I, antennomere II more than twice the length of VI, antennomeres III–V strongly conical (infundibular), VI and VII subrectangular, ultimate disc with 4 apical cones. Tömösváry organ small, located between antennal base and ommatidia, nearly touching the foremost ommatidium. Mandible cardo small (Fig. 1B), stipes ca 3× as long as cardo. Gnathochilarium (Fig. 2D) consisting of a larger eumentum and a smaller promentum, lamellae linguales and long stipites; stipites 2× as long as lamellae linguales and slightly swollen.

***Collum*** not concealing head from above, nearly as wide as head, anteriorly smooth, only posterior third with poorly developed crests (Figs 1B, 2B).

***Body*** cylindrical, with tergites and pleurites fused, sternites free (Fig. 2E). Body rings half as long as wide, 3 penultimate body rings shorter, ca 1/3 as long as wide. Pleurotergites with an inconspicuous median suture, composed of smooth prozonites and carinate metazonites, latter being greater in diameter than prozonites. Prozonites void of crests, with minute scale-like ornamentation (Fig. 1B), metazonites with 28 more or less well-developed longitudinal, narrow, subparallel crests, well-separated from one another, extending over whole body ring, gradually reduced in size laterally and ventrally (Fig. 1D). Crests most pronounced on pleurotergites 3–8. Anterior 4 body rings narrower than following body rings, with less conspicuous crests. Ozopores inconspicuous, an ozopore-like opening visible on body ring 8 (Fig. 1D) and also possibly on body ring 4, situated between the crests, poriferous crests missing. Pleurotergal setae absent.

***Telson*** enlarged, spatulate, 2× the size of the last body ring, dorsal side slightly concave anteriorly (Figs 1E, 2F). Epiproct with inconspicuous crests and 2 spinnerets. Hypoproct divided into a single median plate and 2 lateral plates, all devoid of macrosetae. Paraprocts (anal valves) projecting posteriorly, divided transversally (Fig. 2G).

***Legs*** Anterior leg of body ring 14 0.35 mm long, legs composed of 8 podomeres, relative lengths coxa = trochanter < tarsus 1 = tarsus 2 < femur < tibia = postfemur = prefemur (Fig. 1F). Tarsus 2 with a short claw. Leg 1 and 2 not visibly modified. Some midbody legs with coxal vesicles.

***Male sexual characters*** unknown.

***Female sexual characters*** a pair of long, tubular, apically club-like vulvae behind leg 2 (Fig. 1G); vulvae 0.9 mm long when extruded, apically with long setae. Basal part of vulvae covered by reddish circles (ca 80 µm in diameter) (Fig. 1H). Third pleurotergite slightly enlarged as is typical for adult female callipodidans.

########## Taxonomic remarks.

Several important characters used in the current systematics of Callipodida are unknown in the described specimen, such as the distribution of coxal vesicles on legs in both sexes, as well as male-specific traits such as the shape of gonopods, the presence/absence of modifications on the head and the anterior part of legs and sternites.

## Discussion

### Taxonomic position

*Burmanopetaluminexpectatum* gen. nov. et sp. nov. is the first fossil callipodidan which shows the typical body plan of the order. The presence of 35 body rings, free sternites, pleurotergites with subparallel crests, well-separated from one another, a dorsal midline suture, a telson bearing spinnerets, a tripartite hypoproct and a pair of long retractable vulvae, allow the species to be unequivocally assigned to the order Callipodida. Fossil Callipodida could be confused with the nowadays much more common Cambalidea (Spirostreptida), which are known from 20 specimens in Burmese amber (Wesener and Moritz 2018), but cambalideans have neither a middorsal tergal suture nor spinnerets, both of which are clearly visible in *Burmanopetaluminexpectatum* gen. nov. et sp. nov. Fossil Callipodida might also be confused with species of Stemmiulida, which are known from eight specimens in Burmese amber, both orders showing similar habitus and tergite ornamentation. However, Stemmiulida can be ruled out by the higher number of ommatidia (five vs only two or three in all members of the order), the presence of a long, tubular, vulva attached to coxa 2 (vulvae in Stemmiulida are located between legs 2 and 3, a unique position within Diplopoda, see Silvestri 1916; Enghoff et al. 2015), the clear separation into prozonite and metazonite (absent in Stemmiulida, Hoffman 1982), presence of coxal vesicles, as well as by the divided hypoproct (Enghoff et al. 2015).

The absence of pleurotergal and hypoproctal setae and the presence of an enlarged spatulate telson are characters not observed in any of the extant suborders and families (Table 1) of Callipodida. Therefore, a new suborder and family, Burmanopetalidea suborder nov. and Burmanopetalidae fam. nov., respectively, have been here proposed to accommodate the new species.

**Table 1.** Main differential characters between Burmanopetalidae fam. nov. and the extant families of Callipodida. *Hannibaliuluswilsonae* Shear, Selden & Gall, 2009 from the Triassic of France is also included in the table, although clear apomorphies of the order are not known.

	Burmanopetalidae fam. nov.	* Hannibaliulus wilsonae *	Sinocallipodidae	Callipodidae	Abacionidae	Caspiopetalidae	Dorypetalidae	Schizopetalidae	Paracortinidae	Tynnomatidae
Length	8.2 mm	53–56 mm	40–70 mm	50–70 mm	19–59	28–45 mm	12–50 mm	12–100 mm	32–60 mm	13–50 mm
Number of PTs	35	39–43	55–72	55–65	46–61	53–66	43–54	35–56	50–85	43–88
Antennae	Antennal articles III-V strongly conical (infundibular); 6–7^th^ subrectangular	Unknown	antennal articles generally long; in *S.thai* article VI short and infudibular	Only VIth article infudibular; 7^th^ article conical	Only VIth article infudibular; 7^th^ article conical	V-VIth articles infudibular; 7^th^ article conical	Generally short, article V-VI infundibular (fig. 5 Stoev and Enghoff 2006; Reboleira & Enghoff, 2015-Lusitanipus)	Only Vth article infudibular; VIIth article conical	Only Vth article infudibular; VIIth article conical	V-VIth articles infudibular??; VIIth article conical
Ommatidia	5, well-separated, arranged in two rows	Numerous (at least 10) arranged in subtriangular patch	5–11, arranged in 2–3 rows, in oval shape in others;reduced in some cave species	Numerous, arranged in subtriangular patch	Numerous, arranged in subtriangular patch	Numerous, arranged in subtriangular patch	Numerous, arranged in subtriangular patch	Numerous, arranged in subtriangular patch	Numerous, arranged in subtriangular patch	Numerous, arranged in subtriangular patch; reduced in some cave species
Collum	Smooth, some crests posteriorly	Unknown	Smooth	Smooth	With crests posteriorly	With crests	Smooth or with moderate crests	With crests or smooth	With crests	With crests
Pleurotergal crests	Moderately to poorly developed; narrow, subparallel.	Metazonites of the pleurotergites smooth, with a distinct transverse depression and ventrolateral rebordered flange	Moderately to poorly developed; narrow, subparallel	Missing, instead of crests there are grooves	Well developed; poriferous crests much larger	Well developed; poriferous crests much larger	Moderately to poorly developed	Moderately developed to lacking	Well developed; poriferous crests much larger	Well-developed, poriferous crests more pronounced
Position of ozopores	Between crests	Not detected	Between crests	Between grooves?	On well-developed poriferous crests	On well-developed poriferous crests	At base of crests or on elevated swelling in Cyphocallipodinae	At base of crests	On well-developed poriferous crests	On well-developed poriferous crests
Telson	Enlarged, spatulate, twice the size of the penultimate segment	Enlarged, 1.5 times the size of the penultimate segment in one specimen, posteriorly rounded in both specimens	Normal, approx. the length of penultimate segment	Normal	Normal, approx. the length of penultimate segment	Normal, approx. the length of penultimate segment	Normal, approx. the length of penultimate segment or slightly shorter/ larger	Normal, approx. the length of penultimate segment	Normal, approx. the length of penultimate segment	Normal, approx. the length of penultimate segment
Coxal pouches present on legs	Unknown	Unknown	3–11	3–21	3–13?	3–15	3–13, 3–22 in Cyphocallipodinae	3–16	3–23	3–19, 3–23
Pleurotergal setae	Absent	Unknown	In anterior position on all pleurotergites	In posterior position on all pleurotergites	In anterior and posterior position	In anterior and posterior position	In anterior and posterior position	In anterior and posterior position	In anterior and posterior position	In anterior and posterior position
Female second leg-pair	Normal legs, maybe coxa slightly elongated	Unmodified?	Normal legs	Modified	Modified	Usually modified	Normal or modified	Normal legs, except for *Euxinopetalum*	Modified – two sclerites	Normal or modified
Period	99 million years ago	~242–247.2 million years ago	Extant	Extant	Extant	Extant	Extant	Extant	Extant	Extant

### Size and number of body rings

Extant Callipodida species vary in length from approximately 12 to 100 mm (Enghoff et al. 2015). The species of the East Mediterranean genus *Eurygyrus* C.L. Koch, 1847 (e.g. *E.bilselli* (Verhoeff, 1940) and *E.ochraceus* C.L. Koch, 1847), reach almost 10 cm and are among the largest members of the order. On the other hand, members of the Anatolian and Balkan genera *Euxinopetalum* Hoffman, 1972, *Dorypetalum* Verhoeff, 1900, and *Schizopetalum* Verhoeff, 1900, as well as some North American Tynommatidae, are between 15–20 mm in size. Small size seems to be correlated with a low number of body rings. Callipodida usually develop through teloanamorphosis which means that the addition of body rings stops at a certain stage which is always the same for a given sex of a given species. In some callipodidans, however, it is possible that other types of anamorphosis occur (e.g. hemianamorphosis) (Enghoff et al. 1993). The number of body rings in the order varies between 35 and 88, with the lowest numbers observed in *Schizopetalumkoelbeli* (Verhoeff, 1895) (35-38) and *Euxinopetalumdobatorum* Hoffman, 1973 (38). Both species are also among the smallest members of the order with adults having a length of 15–17.5 mm.

*Burmanopetaluminexpectatum* gen. nov. et sp. nov. is remarkable with a body size in an apparently mature female of just 8.2 mm, which is an extreme case of miniaturization for the order. At the same time the number of body rings is highly reduced. The presence of a pair of long vulvae demonstrates that the specimen is a mature female which most likely reached its last stadium and full body length.

Miniaturisation in fossil Chelicerata has been suggested to be due to the utilization of new ecological niches which larger chelicerates were not able to colonise (Dunlop 2019), which might also be the case here. Simplified eyes and the lack of setation of *B.inexpectatum* gen. nov. et sp. nov. might also be correlated with the general miniaturization of its body and a simplification of the sensory system, rather than with a subterranean manner of living (Dunlop 2019). Furthermore, *B.inexpectatum* gen. nov. et sp. nov. does not show any of the general cave adaptations observable in the Diplopoda (Liu et al. 2017b). Nevertheless, given that the sister order Chordeumatida mostly consists of minute to small species, it is equally possible that the small size of the new species is an ancestral trait.

### Pleurotergal setae

Burmanopetalidae fam. nov. is well characterized by the lack of any pleurotergal setae. Pleurotergal setae are traditionally used as a family- and even subordinal-level character in the classification of Callipodida (Hoffman 1982; Enghoff et al. 2015). In the suborder Callipodidea Pocock, 1894 they are present in a posterior position on all pleurotergites, while in the suborder Sinocallipodidea Shear, 2000 they emerge from the anterior end of the pleurotergites. In the largest callipodidan suborder Schizopetalidea, setae are in an anterior position in the anteriormost pleurotergites, migrating completely to a caudal position usually by the 8^th^ or 9^th^ one (Hoffman 1982). Thus, having a species completely devoid of pleurotergal setae is a unique state, which coupled with several other morphological characters fully justifies the creation of both a new suborder and a new family. We exclude the possibility that the lack of body pilosity is due to taphonomic reasons, as setae seem to preserve generally well in Burmese amber, even in much smaller, 5 mm-long Siphoniulida (Liu et al. 2017a). In addition, the setae on the vulvae are well-preserved in the specimen.

### Telson

The shape of the telson in Callipodida is subtriangular and rather uniform. In most species it is almost equal in size or smaller to the last body ring. In small species up to 15 mm long, i.e. *Dorypetalum*, some Tynnomatidae, it is proportionally reduced. In *B.inexpectatum* gen. nov. et sp. nov., however, the telson is highly enlarged, twice the size of the penultimate body ring and with a spatulate shape. To the best of our knowledge, such a shape is not known in any extant callipodidan. Furthermore, although the hypoproct is subdivided into three plates as in most extant callipodidans, it lacks macrosetae, which are normally present in all extant species in the combination 1+2+1.

### Eyes

The majority of adult callipodidans have eyes composed of at least 30 ommatidia grouped in a subtriangular eye patch (Enghoff et al. 1993). The only exceptions are observed in some cave-dwellers such as species of the North American genus *Tetracion* Hoffman, 1956, as well as the two highly specialized Asian species *Sinocallipussimplipodicus* Zhang, 1993 and *S.jaegeri* Stoev & Enghoff, 2011 (Stoev and Enghoff 2011). However, even in the most cave-adapted taxa, the number of ommatidia in adults is more than 10, while in the surface-dwelling species *B.inexpectatum* gen. nov. et sp. nov., we witness an extreme reduction to only five ommatidia situated in two rows. Juvenile callipodidans usually hatch from eggs with only one ommatidium. Stadium II would have three (1+2), stadium III would have at most six (1+2+3) ommatidia, thus the adult number of five would have been reached at third larval stadium earliest. Some callipodidans (e.g. *Callipusfoetidissimus* (Savi, 1819)) do not add any ommatidia between stadia I and II (Enghoff et al. 1993). Nevertheless, the fully developed cyphopods leave no doubt that the holotype is a fully mature specimen.

### Abundance and distribution

Callipodida is the rarest among all millipede orders preserved in Burmese amber, with only a single specimen out of 529 specimens hitherto known (Wesener and Moritz 2018; Jiang et al. 2019). Even nowadays Callipodida are far less common compared than Julida, Polydesmida, Spirostreptida, Chordeumatida, and Spirobolida, which prevail in temperate and tropical forests. In most cases, being habitat specialists, mostly petrophilic and associated with limestone, callipodidans are usually represented by only a few individuals in the collecting sites (Stoev et al. 2008).

Callipodidans have not previously been recorded from Myanmar (Likhitrakarn et al. 2017), and thus, the finding of *B.inexpectatum* gen. nov. et sp. nov. extends the historical range of the order in Southeast Asia. Of all contemporary families of Callipodida, Burmanopetalidae fam. nov. is geographically closest to Sinocallipodidae Zhang, 1993, which is known from China, Thailand, Laos, and Vietnam (Stoev and Enghoff 2011), Paracortinidae Wang & Zhang, 1993 from China and Vietnam (Stoev and Geoffroy 2004; Liu and Tian 2015), and Caspiopetalidae Lohmander, 1931 from China and Central Asia (Stoev and Enghoff 2005).

### The fossil record

*Burmanopetaluminexpectatum* gen. nov. et sp. nov. can be readily distinguished from *Hannibaliuluswilsonae*Shear et al. 2009, a possibly nematophoran callipodid-like millipede of early Triassic (Anisian) age (ca 243 Mya) by having 'normal' undivided metazonites (vs divided by a wide, shallow transverse depression into anterior and posterior parts, with ventral margins strongly rebordered) and eyes composed of only five ommatidia (vs eyepatches with numerous ommatidia). Furthermore, the Triassic fossil is known to have a body composed of 40–44 body rings and a much longer length (ca 55 mm). Ozopores, pleurotergal setae and spinnerets have not been detected in *H.wilsonae* (Shear et al. 2009).

Enghoff (1990) provided a reconstruction of the ground-plan of the chilognathan millipede based on a cladistic analysis. He argued that the hypothetical ancestor should be regarded as a few-segmented, small animal, lacking trichobothria, with eyes, 8-segmented antennae, defense glands, middorsal suture, segments composed of free sternites and pleurotergites, and a simple telson. *Burmanopetaluminexpectatum* gen. nov. et sp. nov., shows remarkable similarity to the chilognathan ground-plan, especially in the minute size, reduced segmentation, the presence of eyes, a middorsal suture, and ozopores.

Until now, all myriapods known from Burmese amber have been assigned to Recent families and even genera. The monotypic genus *Kachinophilus*Bonato et al., 2014 was recognized as a member of the currently widespread family Geophilidae Leach, 1815 (Bonato et al. 2014). Likewise, the only species of class Symphyla described from Burmese amber, *Symphylellapatrickmuelleri* Moritz & Wesener, 2018, is referred to a genus with more than 40 extant species (Moritz and Wesener 2018). In Diplopoda, the only penicillatan millipede *Polyxenusburmiticus* was assigned to the extant genus *Phryssonotus* in the family Synxenidae (Rasnitsyn and Golovatch 2004). Specimens of Polyxenida recently found in Burmese amber (Wesener and Moritz 2018) were assigned to Polyxenidae and Synxenidae, as well as to an uncertain family. Likewise, the subclass Helminthomorpha Pocock, 1887 was hitherto represented in the fossil record by three species (Liu et al. 2017a; Jiang et al. 2019), all assigned to the contemporary genera *Siphoniulus* Pocock, 1894 and *Siphonophora* Brandt, 1837. In their checklist of the Myriapoda found in Burmese amber Wesener and Moritz (2018) reported only very few taxa that possibly belong to extinct, yet undescribed families. For instance, a number of specimens of the suborder Cambalidea Cook, 1895 were found to possess frontal setae on their head, a character which is not present in recent Spirostreptida. In other terrestrial arthropods with similar habits and evolutionary patterns, numerous specimens from Burmese amber are assigned to families that are no longer extant (for a complete list see Ross 2018). In scorpions alone, there are five families known only as fossils: †Palaeoeuscorpiidae, †Palaeotrilineatidae, †Sucinolourencoidae, †Chaerilobuthidae, and †Palaeoburmesebuthidae, and in the order Ricinulei, all fossil taxa have been assigned to extinct families – †Hirsutisomidae, †Poliocheridae, and †Primoricinuleidae (Ross 2018).

With this detailed description of the first fossil Callipodida from the Mesozoic, we lay down the foundation for further research on the classification and phylogeny of the group. Furthermore, the minimum age of order Callipodida is now known to be at least 99 Mya.

## Supplementary Material

XML Treatment for Burmanopetalidea

XML Treatment for Burmanopetalidae

XML Treatment for Burmanopetalum

XML Treatment for Burmanopetalum inexpectatum
